# Reply to Marakasova and Baranova, “MMR Vaccine and COVID-19: Measles Protein Homology May Contribute to Cross-Reactivity or to Complement Activation Protection”

**DOI:** 10.1128/mBio.03682-20

**Published:** 2021-02-02

**Authors:** Jeffrey E. Gold, David J. Hurley, Balázs Rada, William H. Baumgartl, Larry P. Tilley, Warren E. Licht

**Affiliations:** a World Organization, Watkinsville, Georgia, USA; b College of Veterinary Medicine, University of Georgia, Athens, Georgia, USA; c Nevada Spine Center, Las Vegas, Nevada, USA; d VetMed Consultants, Inc., Santa Fe, New Mexico, USA; e Warren Alpert Medical School of Brown University, Providence, Rhode Island, USA; Albert Einstein College of Medicine

## REPLY

Marakasova and Baranova make a good, incremental step trying to explain the results of our paper, “Analysis of Measles-Mumps-Rubella (MMR) Titers of Recovered COVID-19 Patients” (1). We believe the predictable waning of mumps IgG titers over time allows mumps titers to serve as a proxy measure of overall MMR II vaccine persistence. Even though we observed a significant inverse correlation only with mumps virus titers, any of the components of MMR II alone or in tandem, including measles as Marakasova and Baranova suggest, could be responsible for what we observed. They present a conventional approach to matching protein sequence and structure to structures recognized by antibodies generated during and after vaccination or disease.

The BLAST sequences and structural projections appear rational and likely are a fair representation of proteins on the surfaces of the two viruses. The focus was to indicate possible antibody binding domains for antibodies generated by measles virus as they might impact binding to coronavirus (severe acute respiratory syndrome coronavirus 2 [SARS-CoV-2]). This is possible and may represent the initial step required to impact the disease development indicated by the correlation we saw between MMR II vaccine persistence and coronavirus disease 2019 (COVID-19) severity.

The structures provided would likely represent weak to moderate affinity binding of some antibodies generated by the multivalent vaccine, MMR II. This binding would not provide much activation of local immunity if the weak to moderate affinity antibodies were present at low levels but would likely provide much more activation if a large quantity of low to moderate affinity antibodies were present to yield a high avidity binding situation that would persist long enough to activate monocytes, macrophages, and dendritic cells in the local tissues of the respiratory tract. Epithelial cells also carry FcRn on their surface that can interact with IgG and transport them for intracellular disruption of viral replication (2). Thus, high avidity binding of virus by IgG could “retard and reduce” viral replication if the cross-reactivity predicted by Drs. Markasova and Baranova is correct.

Vaccination, particularly with multiviral live vaccines, tends to produce many different memory clones with a broad range of affinities. In contrast, natural disease, particularly if severely acute, tends to induce somatic mutation in antibody clones that yields very high affinity clones of memory cells of a narrow specificity. This may contribute to the difference seen between those with antibodies from MMR II and natural disease. The narrow specificity may not provide sufficient recognition of the weakly or moderately comparable targets to induce a cascade effect to slow the SARS-CoV-2 infection.

It is possible that measles vaccine-induced T cell memory is partly responsible for the effect leading to the correlation we observed. Only moderate affinity is required for reactivation of T cell memory clones to undergo a few rounds of expansion, leading to production of cytokines and other products that circulate to aid the innate response on local mucosal tissues. The antigen similarity shown in their letter may be sufficient to accomplish this.
